# Altered Resting-State Functional Connectivity in the Default Mode Network in Male Juvenile Violent Offenders

**DOI:** 10.1007/s11682-021-00535-3

**Published:** 2021-09-04

**Authors:** Qiaoling Sun, Yingdong Zhang, Jiansong Zhou, Xiaoping Wang

**Affiliations:** 1 Department of Psychiatry, the Second Xiangya Hospital, Central South University, 139 Middle Renmin Road, Changsha, 410011 Hunan China; 2 China National Clinical Research Center On Mental Disorders (Xiangya), China National Technology Institute On Mental Disorders, Hunan Key Laboratory of Psychiatry and Mental Health, Changsha, 410011 Hunan China; 3 Department of Psychiatry, Hebei Medical University First Affiliated Hospital, Shijiazhuang, 050000 Hebei China

**Keywords:** Juvenile violence, Resting-state fMRI, Default mode network, Functional connectivity

## Abstract

Young males are often associated with more violence, leading to some serious negative consequences. However, the physiology and the neuroimaging patterns underlying juvenile violence remain unclear. Of the limited knowledge on juvenile violence, the default mode network has been known to be associated with its pathophysiology. This study aimed to investigate functional connectivity alterations of the default mode network in male juvenile violent offenders. 31 juvenile violent offenders in a high-security facility, who were convicted of aggressive behaviors by court, and 28 normal controls from a middle school were recruited as participants. They underwent a resting-state functional magnetic resonance imaging scan. And independent component analysis approaches were used to analyze their data. Compared to the normal controls, the juvenile violent offenders showed a different default mode network pattern, with the functional connectivity increased in the posterior cingulate, and decreased in the right middle temporal, left angular, right precuneus and right middle frontal cortex. Our findings revealed that the male juvenile violent offenders were associated with abnormal default mode network functional connectivity, which might be a neuroimaging basis for their tendency to violence.

## Introduction

In recent years, the number of violent crimes has been increasing worldwide (Krug et al., 2002; Mikton et al., 2016), with juveniles and young adults associated with a higher rate of violent crimes than those in other age groups. According to statistics, they are also responsible for a large number of violent crimes (Piquero et al., 2003). It is estimated that the prevalence of juvenile violence worldwide is as high as 30%, which has posed great impact on themselves and the society (Czabański, 2008). Violence involvement in the period of adolescence is associated with more frequent and more serious crimes in the offenders’ life (McDougall et al., 2015), which may further lead to serious negative consequences in their adulthood, such as impaired social relations, lower level of education and increased rate of victimization (Farrington, 2003). It also causes enormous financial burden (Welsh et al., 2008) due to the direct costs of health care and indemnities, as well as indirect costs incurred from declined productivity, injuries and diseases caused by youth violence. According to the global data released by the World Health Organization, the rates of violent crimes by males are significantly higher than those by females in all age groups, with the crime rate among male adolescents (15–29 years old) being the highest (Krug et al., 2002), suggesting that young males are the main group of violent crimes among all age groups. A better understanding of the biological mechanisms underlying violent behaviors in juveniles, therefore, may bring great benefits to the society through providing basis for the identification, management and prevention of violence.

To date, many studies have been directed towards the neurobiology of violence. However, the physiology and the neuroimaging patterns of violence, especially juvenile violence, still remain unclear. According to some studies, biological changes and impairments in some brain regions were related to impulse and violence (Piquero et al., 2003) and might be an important biological factor of violence. In recent years, some neuroscientific studies have found that the brain is more like a huge intricate network and the cognitive, thinking, and controlling functions rely on its comprehensive processing of information (Piquero et al., 2003). As the most important brain network, the default mode network (DMN) is constantly preparing the brain for any upcoming stimulus, thus affecting the perception and response to stimuli (Welsh et al., 2008). Alterations of this network might lead to differences in individual’s sensory and cognitive functions, which can further result in different behavioral responses to the same stimulus (Piquero et al., 2003). The DMN is composed of a group of distant brain regions (Welsh et al., 2008), including the posterior cingulate cortex, precuneus, medial prefrontal cortex and lateral/medial temporal lobes, and plays an essential role in emotional processing, monitoring environment changes, self-introspection, maintaining self-awareness, as well as extracting episodic memories (Piquero et al., 2003). This network is also crucial in the integration of information (Welsh et al., 2008) and has significant functional relation with other areas of the brain. A study suggested that abnormal information integration associated with the DMN is an important factor in the cognition dysfunctions in many disorders (Mckinlay et al., 2013; Rosell et al., 2015). An aberrant activity or connectivity within the DMN might also be associated with abnormal internal reactions to external stimuli, affecting various emotional and behavioral processes (Rosell & Siever, 2015).

Functional magnetic resonance imaging (fMRI) is a useful tool for understanding the neurobiological basis of violence (He, 2014; Meehan & Bressler, 2012). With good functional and spatial resolution, this technology has enabled us to seek to understand the functional connectivity within brain networks. Many studies that assessed brain networks using fMRI have shown that changed functional connectivity of distant brain regions may lead to tendency of violence (Deco et al., 2011; Fox & Raichle, 2007). According to recent literature, violent and non-violent individuals exhibit different baseline neurobiological architecture (Boly et al., 2007), which also involved the DMN (Buckner et al., 2008; Laird et al., 2009). However, most relevant studies have focused on adults (Immordino-Yang et al., 2012; Luo et al., 2011). According to a growing amount of neuroscientific evidence, compared with adult brains, developing brains show important differences in functional organization and activities, such as weaker long-distance connections and stronger short-distance connections in children’s brains (Jacobs et al., 2013). Therefore, through comparing functional brain activities of DMN in juvenile violent offenders with normal controls, we may find clues for why these offenders are more prone to resort to violence, and improve our understanding of the fundamental brain mechanisms underlying violence.

The objective of the present study is to investigate the DMN of male juvenile violent offenders and normal teenagers and explore the abnormal patterns of the DMN in juvenile violent offenders. Based on previous studies, we hypothesized that compared to normal controls, juvenile violent offenders may have a different DMN activity or connectivity pattern.

## Methods

### Participants

Thirty-one male juvenile violent offenders, who were convicted of aggressive behaviors by court, were recruited from the Hunan Provincial Youth Detention Center in China on the basis of the severity of their index offense as recorded by the police. Specifically, all of their crimes were homicide or assault. Twenty-eight normal controls without any history of violent behaviors were recruited from a middle school in Changsha City. All the participants were between 15 and 18 years of age, right-handed (self-reported), and had no history of neurological impairment. All the interviews were conducted by psychiatrists who were informed about the nature of this study. We used the Chinese version of the Schedule for Affective Disorders and Schizophrenia for School-Age Children Present and Lifetime version (K-SADS-PL) (Pessoa, 2008) to assess the participants’ current and lifetime psychiatric problems according to the DSM-IV criteria. The detailed assessment process using K-SADS-PL can be found in our previous study (Zhou et al., 2012).

Information about each participant’s criminal history, psychosocial history, use of alcohol or other drugs, family history, and history of psychiatric and other medical treatments was also collected and recorded. Individuals with any current or lifetime schizophrenia and other psychotic disorders, bipolar disorders, major depressive disorder and the history of substance misuse within the past 3 months were excluded. All the participants were assessed using the Barratt Impulsiveness Scale 11(BIS-11), which consists of 30 items about common impulsive or non-impulsive behaviors and involves three factors: (1) attentional impulsiveness, or the lack of focus on a task engaged; (2) motor impulsiveness, or the ability to inhibit proponent responses; (3) non-planning impulsiveness, or the lack of future planning and forethought (Patton et al., 1995). According to previous data, the score of the scale is correlated with risk behaviors and clinical symptoms (Stanford et al., 2009).

All the subjects and their legal guardians were fully informed of the procedures and signed the written informed consent form. The study was approved by the Biomedical Ethics Board of the Second Xiangya Hospital of Central South University in China.

### MRI Acquisition

Images were obtained with the use of a Siemens 3 T MRI scanner (Siemens Allegra; the Magnetic Resonance Center of Hunan Provincial People’s Hospital, Hunan, China). The following parameters were applied for the functional imaging: repetition time/echo time (TR / TE) = 3000/30 ms, 36 slices, 64 × 64 matrix, 90° flip angle, 240 × 240 mm^2^ FOV, 3 mm slice thickness and no gap. A total of 100 volumes were recorded. Participants were required to simply rest motionlessly in the scanner with their eyes closed during the scanning and not to fall asleep or perform any specific cognitive exercise.

### Data Preprocessing

Data preprocessing was conducted in Matlab (R2013b) using the statistical parametric mapping software package (SPM12, http://www.fil.ion.ucl.ac.uk/spm/software/spm12/). The first five time points were removed to only include MR signals in a steady state. The remaining fMRI data underwent the slice timing, before the head motion was corrected by estimating the values of translation (mm) and rotation (degree) for each participant. More than 2 mm of maximum displacement in x, y, or z axes and 2° of angular motion during the scanning was considered as excessive head movement, and one participant in the offender group was excluded for this reason. The remaining 30 juvenile violent offenders and 28 normal controls were included for further independent component analysis (ICA). The images were normalized to the standard Montreal Neurological Institute (MNI) template, resampled to 3 × 3 × 3 mm^3^, and smoothed with an isotropic Gaussian kernel (full-width at half-maximum = 8 mm) to reduce spatial noise.

### Group ICA and DMN identification

Spatial ICA was performed using the Group ICA in the fMRI Toolbox (GIFT) (http://mialab.mrn.org/software/#gica) to identify independent components (ICs) for all the 58 participants. Dimension estimation on the datasets of the two groups was performed to determine the number of ICs using the minimum description length criterion. 27 ICs were identified and were acquired using the infomax algorithm. Network components were examined visually to eliminate those obvious artifacts. Then the DMN components of the two groups were selected based on the largest spatial correlation with a DMN template offered by GIFT.

### Statistical analysis

Statistical analyses of demographics and clinical data were carried out using the Statistical Package for Social Sciences (SPSS) version 23.0 (SPSS, Chicago, Ill., USA), and the fMRI images were analyzed using the Resting-State fMRI Data Analysis Toolkit (REST) (Wahlund et al., 2009). The normality of continuous data was evaluated using the Kolmogorov– Smirnov one-sample test. Age, education and BIS attentional impulsiveness score did not fit the normal distribution, and these three variables were analyzed with the Mann–Whitney U test. BIS total score and the other two factors was normally distributed, and these variables were analyzed with the two-sample t-test. Chi-squared test or Fisher's Exact Test was used for the analyses of categorical variables. The significance level was set at 0.05 for all the analyses. After the spatial maps of each DMN were extracted from the data of all participants, random-effects analysis using one-sample *t*-tests (*p* < 0.05, with FDR correction) was performed for each group. To explore the DMN differences between the two groups, two-sample *t*-tests was then performed on the individual DMN maps with AlphaSim correction (*p* < 0.05) using the REST software, on basis of the Monte Carlo simulation in AFNI (http://afni.nimh.nih.gov/afni/doc/manual/AlphaSim). A combination threshold of voxels' *p* < 0.001 and cluster size > 351 mm^3^, which corresponded with a corrected *p* < 0.05, was considered significant. Age and education level were included as covariates in the statistical analyses of the present functional data. In addition, Spearman’s correlation was calculated between variations in DMN z values and BIS-11 scores within the offender group. Correlation analysis was performed using SPSS 23.0, with the significance level set at a significance level of *p* = 0.05.

## Results

### Demographics and Clinical Characteristics

The demographic and clinical characteristics of the 30 juvenile violent offenders and 28 normal controls included for analysis of DMN are presented in Tables 1 and 2. According to our analyses, the juvenile violent offenders had a significantly lower education level and family income, a significantly higher rate of smoking, drug and alcohol use, and were approximately one year older than the normal controls. There were also significant differences between groups in BIS-11 total score, scores of all the three BIS-11 factors and the detection rate of conduct disorder. The juvenile violent offenders also had significantly higher scores for the overall BIS-11 scale, motor impulsiveness and non-planning impulsiveness, significantly lower scores in the factor of attentional impulsiveness, compared with the normal controls; they were also associated with higher detection rate in conduct disorder.

**Table 1:** Characteristics of the participants

	Juvenile violent offenders (*n* = 30)	Normal controls (*n* = 28)	Statistical value	*p*
Age (year)	17.03 ± 0.72	16.00 ± 0.38	84.00	< 0.001
Education (year)	7.83 ± 2.07	10.03 ± 0.00	101.00	< 0.001
Smoking, *n* (%)	29 (96.7)	5 (17.9)	37.083	< 0.001
History of drug use, *n* (%)	15 (50.0)	0 (0.0)	18.884	< 0.001
History of alcohol use, *n* (%)	29 (96.7)	21 (75.0)	5.718	0.017
Family income (monthly), *n* (%)^a^			22.209	< 0.001
< 1000	19 (63.3)	3 (10.7)		
1000 - 1999	11 (36.7)	15 (53.6)		
≥2000	0 (0.0)	10 (35.7)		
Parental marital status (divorced), *n* (%)	4 (13.3)	2 (7.1)	0.598	0.439
Main guardian, *n* (%)			0.308	0.857
Parents	27 (90.0)	24 (85.7)		
Grandparents	2 (6.7)	3 (10.7)		
Other	1 (3.3)	1 (3.6)		
Family type, *n* (%)			0.474	0.925
Extended family	4 (13.3)	4 (14.3)		
Core family	23 (76.7)	20 (71.4)		
Single-parent family	1 (3.3)	2 (7.1)		
Not living with parents	2 (6.7)	2 (7.1)		
Type of crime, *n*				
Homicide	3	-		
Intentional injury	14	-		
Robbing and causing more than minor injuries	12	-		
Rape and cause minor injuries	1	-		
Duration of penalty (months), median (IQR)	90(57-123)	-		
Time served (months), median (IQR)	18(12-20)	-		

^a^ note: Chinese Yuan (CNY), minimum monthly salary (per person); n: number; IQR: interquartile range

**Table 2:** Descriptive statistics for impulsiveness (BIS-11) and K-SADS-PL

		Juvenile violent offenders (n = 30)	Normal controls (*n* = 28)	Statistical value	*p*
BIS					
Total score		72.47$\pm$4.74	64.57$\pm$5.54	5.844	< 0.001
Attentional impulsiveness		19.40$\pm$3.07	22.14$\pm$2.41	185.500	< 0.001
Motor impulsiveness		24.20$\pm$2.98	21.43$\pm$3.34	3.342	0.001
Non-planning impulsiveness		28.87$\pm$3.35	21.00$\pm$2.09	10.804	< 0.001
Diagnosis of any disruptive behavioral disorder, *n*					
Attention deficit Hyperactivity disorder		2	0	1.933	0.492
Oppositional defiance disorder		7	0	7.430	0.11
Conduct disorder		24	1	34.496	< 0.001
Previous diagnosis of alcohol abuse, n^b^		3	0	2.953	0.238
Previous diagnosis of drug abuse, n^b^		5	0	5.107	0.053

^b^ Note: They had not been off drugs or alcohol for at least ten months

### Intra-Group ICA Analyses

**Fig. 1:**
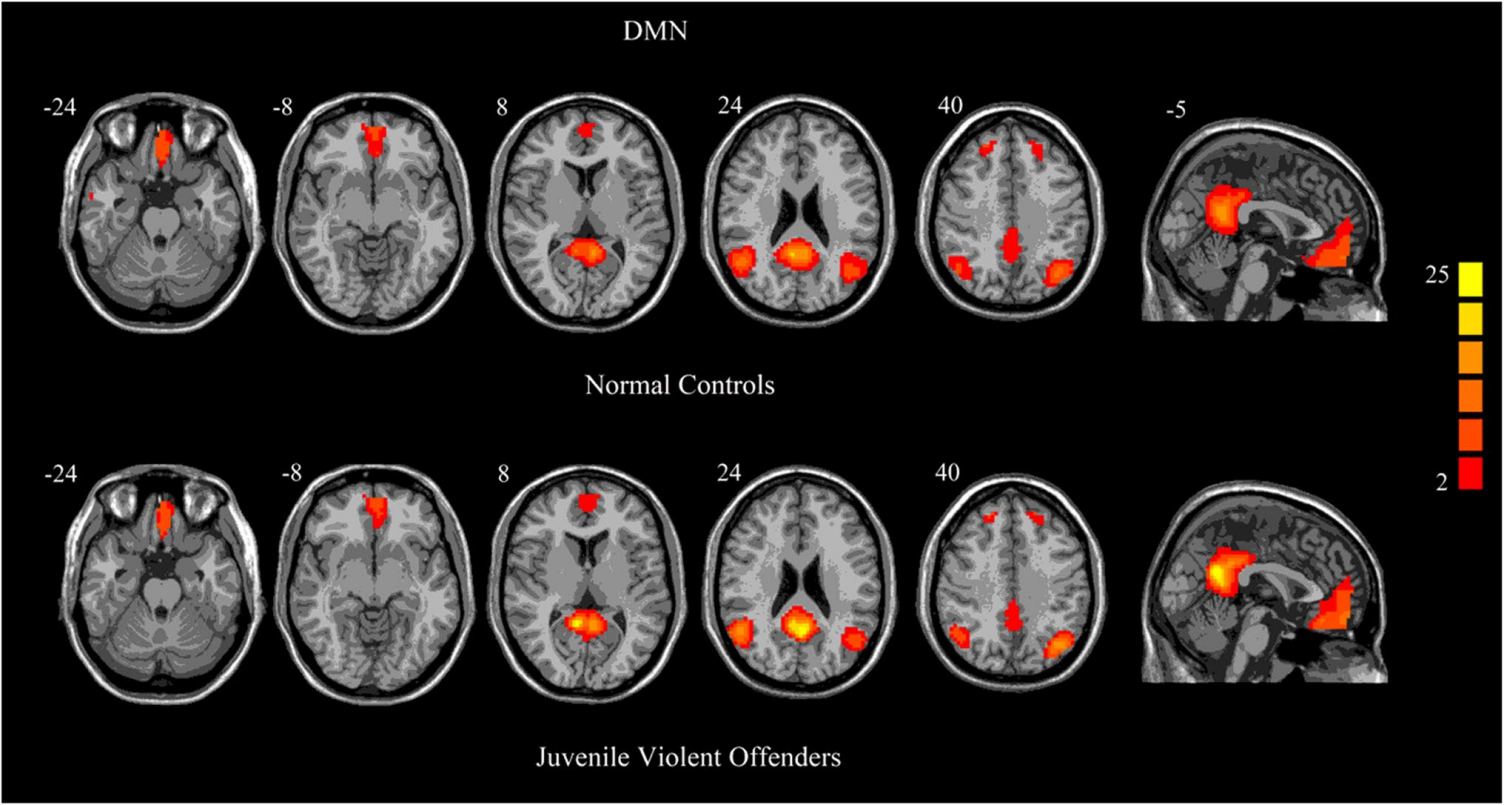
One-sample t-test results for group-level DMN in normal controls and juvenile violent offenders. *p* < 0.05, FDR corrected

The one-sample *t*-tests revealed a typical spatial pattern in the DMN in the two groups (Fig. 1), which included brain regions such as the bilateral medial prefrontal cortex, the posterior cingulate cortex/precuneus, anterior cingulate cortex, bilateral angular gyri and middle temporal gyrus.

### Inter-Group ICA Analyses

**Fig. 2:**
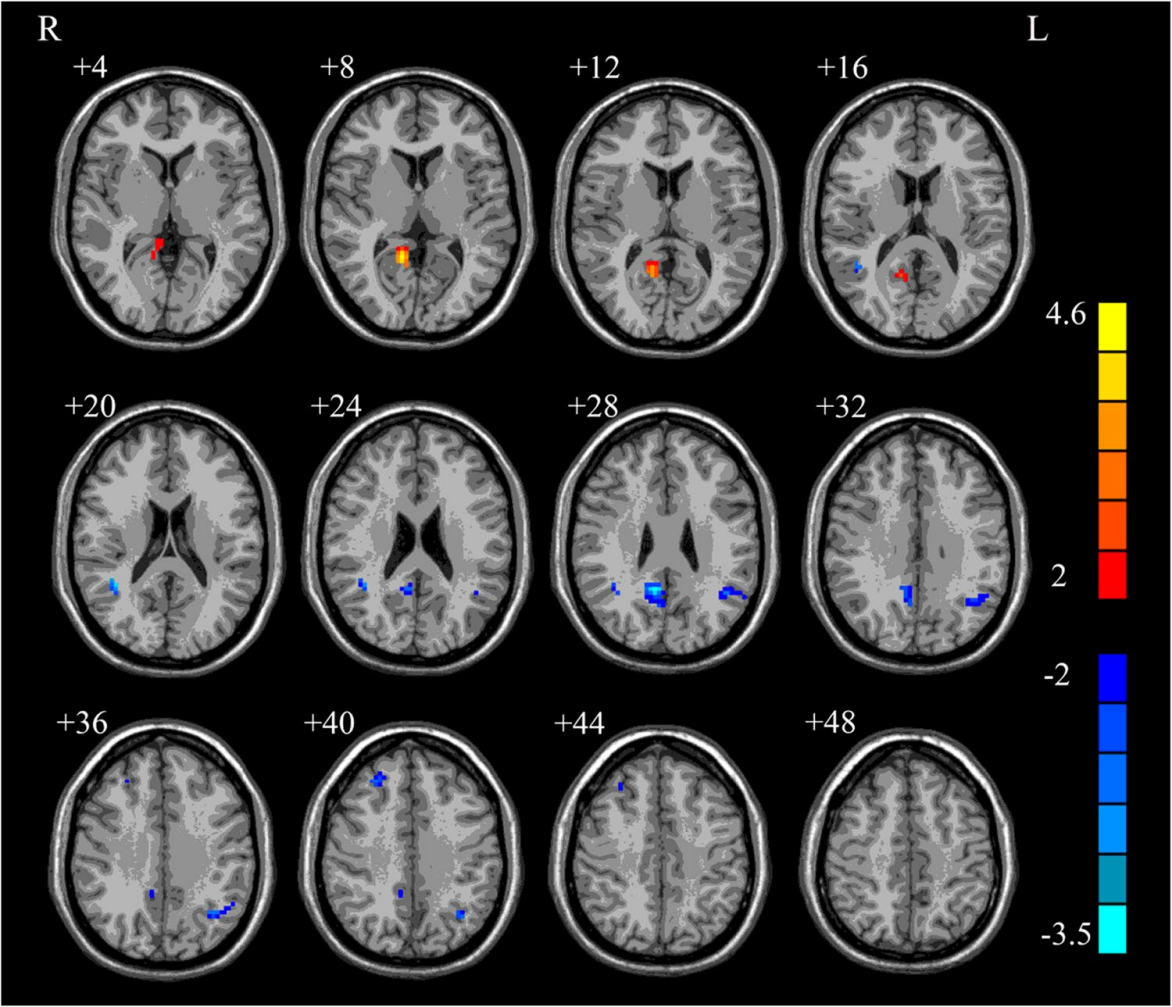
Brain regions with significantly altered connectivity within DMN in juvenile violent offenders compared to normal controls. *p* < 0.05 with AlphaSim correction

The results of two-sample *t*-tests showed significant differences in DMN between the two groups (*p* < 0.05, AlphaSim correction). The connectivity differences were found in the posterior cingulate, left angular, right precuneus, right middle frontal and right middle temporal (Fig. 2). The regions displaying DMN connectivity differences, along with the MNI coordinates of the peak foci, were listed in Table 3. Compared with the normal controls, the offenders showed increased functional connectivity in the posterior cingulate, and decreased functional connectivity in the right middle temporal, left angular, right precuneus and right middle frontal.

**Table 3:** Abnormal DMN regions in male juvenile violent offenders relative to normal controls. *p* < 0.05 with AlphaSim correction

Region	Peak (MNI)			T value	Cluster size
	x	y	z		
juvenile violent offenders > normal controls					
Posterior cingulate	12	-51	9	4.147	51
juvenile violent offenders < normal controls					
Right middle temporal	39	-48	21	-3.048	23
Left angular	-42	-54	30	-2.784	51
Right precuneus	9	-51	30	-3.451	63
Right middle frontal	27	36	39	-2.506	20

### Correlation Analysis of the Offender Group

No correlation was found between the BIS-11 total score or scores of three factors and the connectivity in the right middle frontal, right precuneus, left angular, right middle temporal and posterior cingulate in the juvenile violent offenders.

## Discussion

This study revealed altered resting-state DMN in juvenile violent offenders using ICA methodology. Compared with the normal controls, the offender group showed increased functional connectivity in the posterior cingulate and decreased functional connectivity in the right middle temporal, left angular, right precuneus and right middle frontal cortex.

The results showed the juvenile violent offenders had altered functional connectivity in the right middle temporal and frontal cortex, which was in line with prior studies that uncovered dysfunctions of frontal and temporal regions in violent populations. Key regions, including the temporal cortex and frontal cortex, are structurally and functionally impaired in the population with antisocial behaviors (Rosell & Siever, 2015). Our previous study found that juvenile violent offenders showed significantly lower regional homogeneity values in the right medial prefrontal cortex than the controls (Chen et al., 2015). In addition, abnormalities of frontal cortex were also found in a group of patients with mental disorders who had a history of violence (Raichle et al., 2001; Shannon et al., 2011), as well as in some high-risk violent offenders (Fairchild et al., 2013; Pardini et al., 2014).

The right middle temporal and frontal area displayed lower functional connectivity in the juvenile violent offenders. One possible reason is that there are potential neurobiological differences in the DMN. Recent neurobiological studies support the idea that abnormalities in emotion-related brain areas may allow individuals to engage in aggressive acts (Yang et al., 2005). Previous studies have shown that the activation of fronto-temporal structures was related to the control over emotion-related behaviors and self-regulation. Patients with impulsive aggressive personality disorder were also found to have impaired emotion regulation and blunted prefrontal area (Leutgeb et al., 2015). Besides, functional abnormalities of certain regions of the frontal area, especially the orbitofrontal region, were related to poor inhibitory control, which might be the reason for excessive violent responses (Klasen et al., 2018). The frontal and temporal lobe play an essential role in regulating emotional and social behaviors (Kuroki et al., 2017), and are important for understanding the emotional of others (Yang et al., 2008), integrating moral knowledge with emotional cues (Schug & Raine, 2009) and inhibiting aggressive impulses (Raine et al., 1997). Individuals with damages in these regions showed inadequate control of emotions as well as poor awareness of their own unsatisfying decision making (Lindberg et al., 2005). In this study, the offender group was associated with higher rate of inappropriate behaviors (such as smoking, drug and alcohol misuse) and higher detection rate of conduct disorder, which was consistent with the above viewpoints. In psychopathy, integrated functioning of the medial orbitofrontal cortex and amygdala in decision-making and stimulus-reinforcement learning is disrupted, which was considered to be the basis of the deficits in appropriate decision-making and socialization (Glenn & Raine, 2009). Aa a supplement for prior works, our study has revealed the association between violence and the disruption in fronto-temporal activity.

The posterior cingulate, precuneus and angular are key DMN hubs. A study found that the posterior cingulate was highly connected with many cortical and subcortical regions, such as medial temporal structures and the medial prefrontal cortex (New et al., 2002). In addition to structural studies that emphasized the important role of the posterior cingulate in brain organization, analyses on glucose metabolism showed that the posterior cingulate has a very high metabolic rate (Izquierdo, 2005; Rudebeck et al., 2008). The blood flow of the posterior cingulate and adjacent precuneus was 40% more than the average quantity (Vollm et al., 2006). The posterior cingulate, with such a hub-like organization, is particularly important for arousal and attention, and controls the balance between internally and externally focused thoughts (Moll et al., 2002). The high connectivity of posterior cingulate may indicate an increased level of arousal and attention on external events, which might explain the lower attentional impulsiveness in the present study. The current work showed lower connectivity in the right precuneus and left angular. In our previous study, lower regional homogeneity values in precuneus were found in violent offenders, compared with the controls (Brower & Price, 2001). A coordinate-based meta-analysis showed altered activation in the right precuneus in aggressive individuals, compared with non-aggressive individuals, under different fMRI paradigms. This abnormality may further disrupt DMN and other neural networks, resulting in failure to generate adaptive responses when needed (Bechara, 2004). In male veterans with traumatic brain injuries, decreased functional connectivity between the angular region and the orbitofrontal cortex was associated with higher physical aggression score (Blair, 2007). The angular gyrus is considered to be one of the component of the brain network regarding moral reasoning (Hagmann et al., 2008), and is also essential in linking emotional experiences to moral appraisals (Raichle et al., 2001). These results suggest that the altered connectivity of the posterior cingulate, precuneus and angular regions might make male teenagers more prone to violence, and also highlighted that the aberrant DMN connectivity might represent a potential trait characteristic for juvenile violence.

In developing children, a correlation between impulsivity and functional connectivity in posterior DMN clusters was found (Inuggi et al., 2014). And the balance between the default network and attention and control networks may affect the impulsivity of individuals (Shannon et al., 2011). However, we did not find any correlation between the BIS-11 total score or scores of three factors and the connectivity in the right middle frontal, right precuneus, left angular, right middle temporal and posterior cingulate of DMN in the juvenile violent offenders. This may be due to the different tools used to measure impulsivity or that DMN clusters closely related impulsivity are not the above-mentioned.

There are several limitations in this study. Firstly, juvenile violent offenders had a shorter period of education, and they were one year older than the controls, which might affect our interpretation as well as the generalizability of the findings. At the stage of research design, we set a narrow age range of 15 to 18 years; but unfortunately, the age of the two groups did not match well. However, previous studies showed that the DMN matures into an “adult-like” network by the age of two (Gao et al., 2011; Keunen et al., 2017). It can be reliably identified in children and adolescents through long-term repeated measures with an interval of 2.5 years (Thomason et al., 2011). Therefore, by adolescence, the spatially distributed and functionally linked DMN has almost reached its adult state. Secondly, only males were included in this study in order to avoid the influence of sex on DMN activation patterns, which precluded us from obtaining relevant findings in females. In addition, it should be considered that aggression is a very heterogeneous construct (Vogt & Laureys, 2005), which might be an important source of variability in brain activation among offenders. However, offenders in this study had committed crimes like armed robbery, assault, murder, or even a combination of those. This may limit the value of the results.

In conclusion, compared with the normal controls, the male juvenile violent offenders showed a different DMN pattern, including increased functional connectivity in the posterior cingulate and decreased functional connectivity in the right middle temporal, left angular, right precuneus and right middle frontal cortex. Abnormalities in these regions may lead to dysfunctions in regulating aggressive behaviors, such as inhibitory control deficits in emotional contexts. Therefore, we proposed that abnormal DMN in the resting-state might contribute to aggressive behaviors in juvenile violent offenders.
